# A Pure Inorganic ZnO-Co_3_O_4_ Overlapped Membrane for Efficient Oil/Water Emulsions Separation

**DOI:** 10.1038/srep09688

**Published:** 2015-04-22

**Authors:** Na Liu, Xin Lin, Weifeng Zhang, Yingze Cao, Yuning Chen, Lin Feng, Yen Wei

**Affiliations:** 1 Department of Chemistry, Tsinghua University, Beijing 100084, P. R. China

## Abstract

The earth's environmental problems, especially for water remediation, need effective methods to solve. Materials with special wettability are developed for the separation of oil/water mixtures. However, the separation of emulsified oil/water mixtures can be a real challenge. There is still much deficiencies, on account of the surfactant, which could link water molecules and oil molecules to form a stabilized system. Here we report a pure inorganic ZnO-Co_3_O_4_ overlapped membrane to give a brand new solution to emulsified oil/water mixture separation. Fabricated by an easy and cost-efficient way, such a membrane combines the properties of under-water superoleophobicity and under-oil superhydrophobicity, which can be successfully used for the efficient separation of both surfactant-free and surfactant-stabilized emulsions, solely driven by gravity. This ZnO-Co_3_O_4_ overlapped membrane shows great potential applications to industrial wastewater treatment, domestic sewage purification and other water remediation.

Efficient processes for oil/water separation, especially for emulsified oil/water mixtures separation are always difficult and still challenging^1^,^2^,^3^. Traditional filtration membranes such as ultrafiltration membranes have been commonly recognized as ideal materials for the separation of various emulsions including surfactant-stabilized emulsions^4^,^5^,^6^,^7^,^8^. Nevertheless, due to the defects of low flux and severe fouling on account of surfactant absorption and/or pore plugging by oil droplets, the performance of filtration membranes is strictly limited and far less than application requirements^9^,^10^.

Superwetting materials fabricated via rational combination of surface composition and rough structure have been applied to oil/water separation^11^,^12^,^13^,^14^,^15^,^16^. Although superhydrophobic^17^,^18^,^19^,^20^,^21^,^22^,^23^,^24^,^25^,^26^,^27^,^28^,^29^ or superoleophobic materials^30^,^31^,^32^,^33^,^34^,^35^,^36^ have been extensively explored, they are limited to separate immiscible oil/water mixtures and become invalid for emulsions especially in the presence of surfactant. Recently, a new breakthrough^12^,^35^, which takes advantage of superhydrophilic and underwater superoleophobic properties of a fluorodecyl POSS + x-PEGDA blend-coated hygro-responsive mesh membranes, has been first proposed by the group of A. Tuteja and used to separate oil/water emulsions with droplet sizes larger than 1 μm. Subsequence, Jin and co-workers fabricated poly (vinylidene fluoride) (PVDF) membrane^37^ and poly (acrylic acid)-grafted PVDF (PAA-g-PVDF) membrane^1^ via salt-induced phase inversion processes, which were conditioned to treat water-in-oil emulsions and oil-in-water emulsions, respectively. To deal with both oil-in-water and water-in-oil emulsions, Liu et al. obtained superhydrophilic-superoleophilic PVDF membrane^38^, but the membranes were unstable when exposed to some harsh conditions such as high temperature. Besides, some works have reported the fabrication of membranes for ultrafast separating emulsions^39^,^40^,^41^,^42^. Nevertheless, an extra pressure generated by a vacuum driven filtration system is inevitable to guarantee the successful operation of these membranes, resulting in the great cost consumption. Therefore, a demand for an inexpensive and universal methodology about designing pure inorganic superwetting materials to efficiently separate various emulsions, especially surfactant-stabilized emulsions solely driven by gravity, is highly desired.

Herein, we report the fabrication of pure inorganic ZnO-Co_3_O_4_ overlapped membrane via an easy, cost-efficient and environmentally friendly hydrothermal method (Fig. 1, left). As schematically shown in Fig. 1 (middle), the membrane displays under-water superoleophobicity and under-oil superhydrophobicity at the same time. Compared to the polymer-dominated filtration membranes that were carried out under external applied trans-membrane pressure, this inorganic overlapped membrane can efficiently separate, for the first time, both surfactant-free and surfactant-stabilized emulsions solely driven by gravity with high flux. More importantly, it exhibits prominent performance in treating oil-in-water and water-in-oil emulsions with high separation efficiency (Fig. 1, right), excellent antifouling property, superior thermal stability as well as long-term use. These merits endow the membrane with the promising applications in fuel purification, emulsified wastewater treatment in industry and daily life.

## Results

### Morphology and Phase Composition

**Figure 2:**
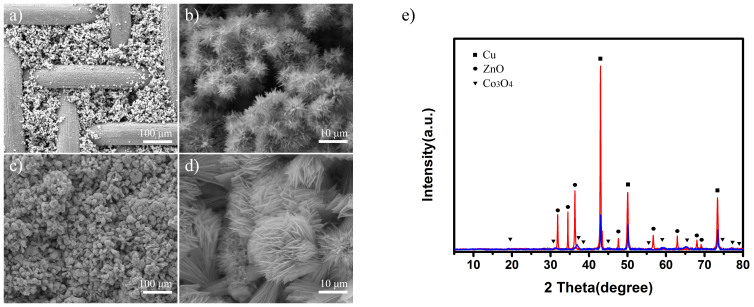
Morphology and XRD results. (a–d) SEM images of ZnO micro-clusters prepared on Cu substrate and ZnO-Co_3_O_4_ overlapped membrane with different magnifications. (e) XRD patterns of the as-prepared membrane.

The commercial copper mesh with the pore diameter of approximately 50 μm was used as the substrate, and its scanning electron microscopy (SEM) image was shown in Supplementary Fig. 1a. In order to increase the surface roughness for better growth of metal oxides on the substrate, Cu electro-deposition was applied and the rectangular pyramid shape of electrodeposited copper could be observed clearly (Supplementary Fig. 1b). Fig. 2a gives the SEM image of ZnO micro-clusters prepared on the electrodeposited Cu substrate. It is obvious that the mesh pores are filled with ZnO. The enlarged image indicates that a large amount of pine needle-like ZnO of several micrometers scale aggregate together to form cluster structures (Fig. 2b). Fig. 2c, d present the SEM images of ZnO-Co_3_O_4_ overlapped membrane at different magnifications after hydrothermal growth of Co_3_O_4_ on the surface of ZnO stuffed mesh. The membrane is mainly dominated by flower-like Co_3_O_4_, which is uniformly distributed and covered on the mesh surface. It is worth noting that the flower-like aggregates of micrometer scale are composed of lamellar structures of nanometer scale. According to Cassie model derived from Young's Equation, these micro- and nanoscale hierarchical structures are crucial for obtaining superwetting feature.

X-ray diffraction experiment is carried out to confirm the phase compositions. Fig. 2e revealed the phase purity of the ZnO-Co_3_O_4_ structures. It is observed that the ZnO diffraction peaks are relatively weak, which is probably due to the limited amount of ZnO deposited on the copper substrate. Similarly, the diffraction peaks of Co_3_O_4_, which obtained by subsequent hydrothermal process, are even weaker than ZnO. Nevertheless, all ZnO and Co_3_O_4_ diffraction peaks can be clearly seen and match up well with the JCPDS cards, which confirm the formation of pure ZnO and Co_3_O_4_. Besides, the strong Cu diffraction peaks are assigned to octahedral Cu derived from electro-deposition.

Through a rough test, the ZnO filled mesh was confirmed to be invalid for separating surfactant-free toluene-in-water emulsion, which is mainly due that the ZnO clusters merely stuff the mesh pores and are easily flushed away by the poured emulsion (Supplementary Fig. 2a). Although flower-like Co_3_O_4_ aggregates grown directly on copper mesh could attach on the mesh firmly, they do not occupy the mesh pores to achieve demulsification (Supplementary Fig. 2b). Other metal oxides such as TiO_2_ and so on, are restricted to their harsh growth conditions as well as failure of covering throughout mesh surface (Supplementary Fig. 2c). Therefore, integration of ZnO and Co_3_O_4_ via successive hydrothermal growth was considered to be an optimal choice to separate oil/water emulsions efficiently.

### Wetting Behavior

**Figure 3:**
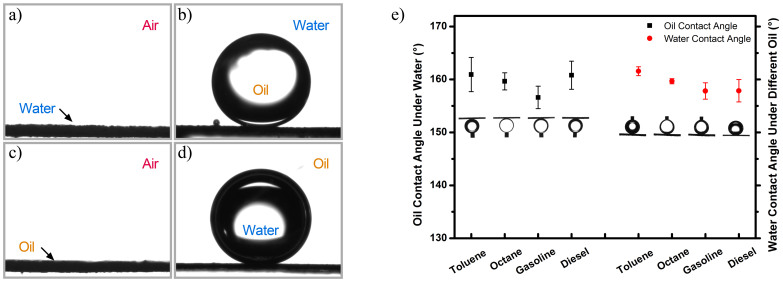
Wetting behavior. (a, b) Wetting behavior of the membrane toward water in air and oil in water. During the under-water oil CA measurement, the membrane was immersed in water and the oil droplet (5 μL) was 1,2-dichloroethane (heavier oil was selected as detecting probe so that oil droplet could fall down and stand on the membrane). (c, d) Wetting behavior of the membrane toward oil in air and water in oil. The water droplet was 5 μL and diesel was used as surrounding condition to measure the under-oil water CA (lighter oil was chosen to allow water droplet to fall down and stand on the membrane). (e) Superwetting behavior of the membrane in oil/water/solid systems for various oils under water and water in different oils.

Owing to multi-scale hierarchical structures in combination with high surface energy composition, the as-prepared ZnO-Co_3_O_4_ membrane exhibits superamphiphilicity and the wetting behavior of water and oil on the as-prepared membrane are shown in Fig. 3a, c. When a water or oil droplet (5 μL) touches to the membrane surface, it quickly spreads out and a nearly 0° contact angle (CA) is obtained. Therewith, the membrane is immersed in water and the oil (1, 2-dichloroethane) wettability is characterized by measuring the under-water oil CAs. The membrane exhibits superoleophobicity with oil CA of 159.2 ± 1.3° (Fig. 3b). Water can be infiltrated and trapped into the rough structure because of inorganic oxides' high surface energy, resulting in great decrease of contact area when oil droplet contacting with the membrane. Therefore, the membrane shows underwater superoleophobic property in an oil/water/solid system. Analogously, if submerged in oil (diesel), because of superoleophilicity, the trapped oil will effectively prevent water droplet contacting with the membrane surface, and the under-oil superhydrophobicity is demonstrated with the water CA of 157.9 ± 2.1° (Fig. 3d). Besides, the CAs of various oils underwater and water under different oils were measured, as shown in Fig. 3e. All the oil/water CAs are larger than 150°, which exhibit superwetting properties of the as-prepared membrane.

### Separation Capability

**Figure 4:**
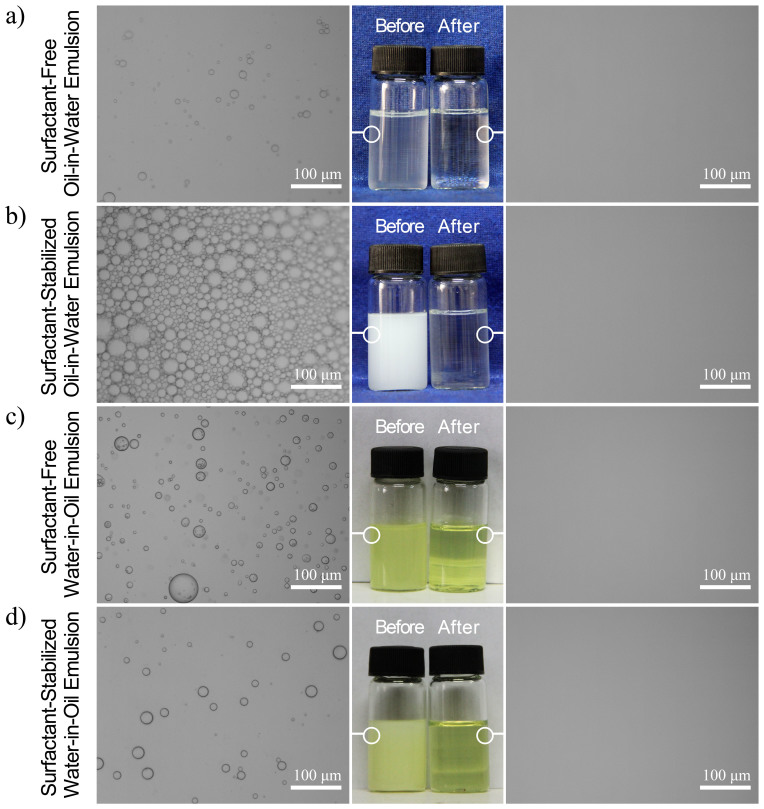
Separation results of various emulsions. (a) Tween20-free diesel-in-water emulsion, (b) tween20-stabilized diesel-in-water emulsion, (c) span80-free water-in-diesel emulsion and (d) span80-stabilized water-in-diesel emulsion.

The particular wettability of the membrane achieved in above two situations is considered to be advantageous to separate oil/water emulsion. To test the separation capability of the membrane, a series of emulsions in different types, including surfactant-free/surfactant-stabilized oil-in-water and water-in-oil emulsions were prepared. The as-prepared emulsions were poured onto the membrane to carry out separation driven by gravity solely and the separation device was shown in Figure S3. Figure 4 gives the separation results of the tween20-free/tween20-stabilized diesel-in-water emulsions and span80-free/span80-stabilized water-in-diesel emulsions as examples, respectively. As shown in the optical microscopy images in either case, compact droplets flood in the feeds of emulsions before permeation, whereas no any droplets are observed in the whole view of the corresponding filtrates, indicating that all diesel/water emulsions have been successfully separated with diesel or water being retained above.

### Permeability and Mechanism

**Figure 5:**
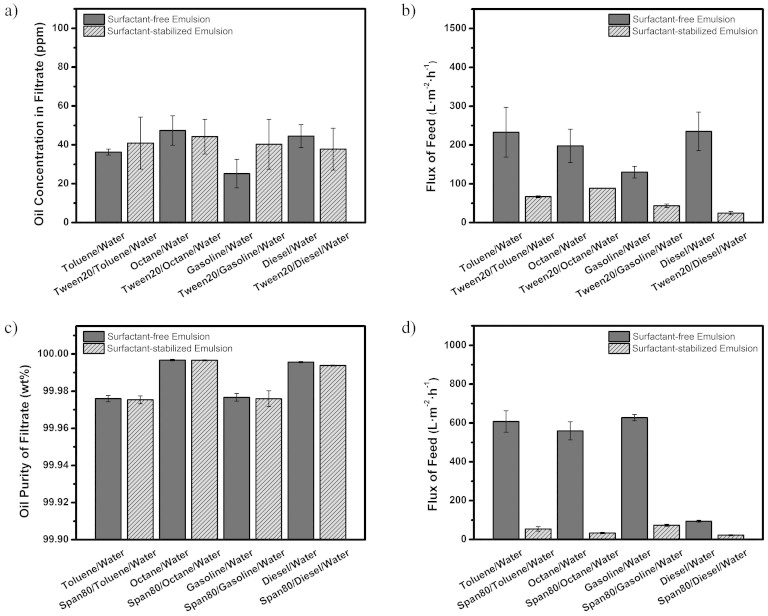
Permeability of the membrane. (a) Oil concentration in the corresponding filtrates and (b) permeation flux of the membrane for various surfactant-free and surfactant-stabilized oil-in-water emulsions. (c) Oil purity of the corresponding filtrate and (d) permeation flux of the membrane for a series of surfactant-free and surfactant-stabilized water-in-oil emulsions.

Membrane permeability was deeply investigated to proof that the inorganic overlapped membrane is appropriate for separating sundry emulsions no matter water is continuous phase or dispersed phase. With regard to oil-in-water emulsions, the oil concentration in collected filtrates was measured by the infrared spectrometer oil content analyzer, which aims to qualitatively analyzing the infrared absorption spectra of oils and accurately measuring the concentrations of oil contaminants. As shown in Fig. 5a, the oil concentrations in the filtrates are all below 50 ppm for all the oil-in-water emulsions. The high separation efficiency of the membrane is probably due to the invasion of continuous water phase, thus its superhydrophilicity and underwater superoleophobicity permit water passing through quickly while oil is blocked on the membrane. The permeation flux of the membrane is calculated by recording the time needed for collecting a certain volume of various oil-in-water emulsions and demonstrated in Fig. 5b. The flux is in the range of 115 ~ 300 L.m^−2^h^−1^ for tween20-free oil-in-water emulsions. For tween20-stabilized emulsions, the values are smaller than those of tween20-free emulsions and pure water, which are mainly attributed to the disturbance of demulsification (Supplementary Tab. 1 and Tab. 2, left). For water-in-oil emulsions, the oil purity of filtrates was examined using a Karl Fischer titrator. Fig. 5c gives the oil purity for all collected filtrates is above 99.97%, indicating an extremely high separation efficiency of the membrane. Interestingly, the “water-repellency” property plays a crucial role in the continuous oil situation here, which guarantees the permeation of oil and efficient obstruction of water. Similar flux of the membrane for sundry water-in-oil emulsions and pure oils are measured and shown in Fig. 5d and Supplementary Tab. 2, right. The flux for water-in-diesel emulsions is lower than those of other emulsions, which is owing to the high viscosity of diesel as fluid viscosity is usually inversely proportional to the flux. Although the superwetting PVDF membranes reported previously have even higher flux, it is the first time that our membrane successfully realizes the fast separation of various oil-in-water and water-in-oil emulsions based on inorganic overlapped materials.

### Reusability and Thermal Stability

The membrane demonstrates superior long-term usability and antifouling property, which are tested using environmental scanning electron microscope. The same membrane is used repeatedly throughout the above emulsion separation experiments. In every cycle after one filtration, the membrane is simply rinsed by little acetone and then dried. Over fifty repetitions, the membrane still keeps stable performance without visible morphology variation (Supplementary Fig. 4). Besides, the thermal stability of our membrane is examined by measuring the under-water oil CA and under-oil water CA after heating it at 300°C for 3 h. As shown in Supplementary Fig. 5, the membrane maintains its oil-repellency and water-repellency with under-water oil CA of 161.6 ± 4.5° and under-oil water CA of 164.1 ± 0.6°. These results play an important role in numerous applications.

In conclusion, we have developed a universal and cost-effective method for fabricating superhydrophilic-superoleophilic ZnO-Co_3_O_4_ overlapped membrane, which can efficiently separate both surfactant-free and surfactant-stabilized emulsions. For the first time, this inorganic membrane displays extremely high separation efficiency with less than 50 ppm oil concentration in filtrates for oil-in-water emulsions and >99.97% oil purity after filtration of water-in-oil emulsions driven by gravity solely. More importantly, its excellent antifouling property, splendid thermal and mechanical stability and ease to cycle endow the membrane with great potential in practical applications for fuel purification, treating wastewater produced in industry and daily life.

## Methods

### Fabrication of ZnO-Co_3_O_4_ Overlapped Membrane

The superhydrophilic-superoleophilic membrane was prepared using a hydrothermal method. Briefly, the copper mesh with pore size approximately 50 μm as substrate was ultrasonically cleaned in deionized water and acetone to remove the surface dirt, respectively. Cu electro-deposition was carried out on mesh surface at a constant potential (1.5 V) for 60 s twice at room temperature^43^, where the electrolyte was an aqueous solution of 0.5 M CuSO_4_. Both the working electrode and cathode were clean copper meshes. After the deposition, the samples were rinsed with deionized water and dried. The 40 mL solution of 0.035 M Zn(Ac)_2_ and 0.55 M NaOH was obtained by magnetically stirring for 15 min^44^. After transferred to a Teflon autoclave, the roughened mesh was carefully immersed in the solution and the sealed autoclave was heated at 90°C. After that, the sample with a white ZnO layer was rinsed with deionized water. Subsequently, 1.164 g Co(NO_3_)_2_.6H_2_O and 1.2 g CO(NH_2_)_2_ were dissolved in 40 mL of water under stirring^45^. After stirring for 15 min, the purple solution and the ZnO deposited mesh were transferred into the autoclave, which was sealed and maintained at 90°C. The aubergine sample was picked out, rinsed with deionized water and dried. Finally, the ZnO-Co_3_O_4_ overlapped membrane was obtained by annealing at 300°C for 3 h.

### Preparation of Oil-in-Water and Water-in-Oil Emulsions

Surfactant-free emulsions were prepared by mixing water and oil (toluene, n-octane, gasoline and diesel, respectively) in 100:1 or 1:100 (v/v) and the mixtures were sonicated for 1.5 h to obtain homogeneous and milky oil-in-water and water-in-oil emulsions. Surfactant-stabilized oil-in-water emulsions were prepared by mixing water and oil (toluene, n-octane, gasoline and diesel, respectively) in 100:1 (v/v) with addition of 0.5 mg of Tween20 per mL of emulsion (0.05 wt%) under high stirring for 1.5 h. Analogously, Surfactant-stabilized water-in-oil emulsions were prepared by mixing water and oil (toluene, n-octane, gasoline and diesel, respectively) in 1:100 (v/v) with addition of 2 mg of Span80 per mL of emulsion (0.2 wt%) under high stirring for 1.5 h. Typically, all the prepared emulsions are stable for more than 3 days without demulsification or precipitation observed when placed in ambient conditions.

### Instruments and Characterization

SEM images were obtained on an environmental scanning electron microscope (FEI Quanta200, Czech). XRD patterns of the samples were recorded on a polycrystalline X-ray diffractometer with a Cu K_α_ radiation source (Bruker D8 Advance, Bruker-AXS, Germany). Contact angles were measured on a contact angle measurement machine (OCA15 machine, Data-Physics, Germany). The surfactant-free emulsions were prepared by sonicating the oil/water mixtures in a numerical control ultrasonic cleaner (KQ-250DE, China). Optical microscopy images were taken on a polarizing microscope (Nikon ECLIPSE LV100POL, Japan). The oil content in the filtrate was measured by the infrared spectrometer oil content analyzer (Oil480, Beijing Chinainvent Instrument Tech. Co. Ltd., China). The water concentration in the filtrate was examined using a Karl Fischer titrator (Cou-Lo Aquamax KF Moisture Meter, UK).

### Emulsion Separation Experiments

The as-prepared ZnO-Co_3_O_4_ overlapped membrane was fixed between two Teflon fixtures. Both of the fixtures were attached with glass tubes and placed vertically. The freshly obtained emulsions were poured onto the membrane and spontaneously separated quickly. The fluxes were determined by calculating the needed time that collected a certain amount of filtrate.

## Author Contributions

L.F. and Y.W. supervised the work. W.Z., Y.C. and Y.C engaged in the data collection and the discussion.

## Supplementary Material

Supplementary Information Supplementary Information

## Figures and Tables

**Figure 1:**
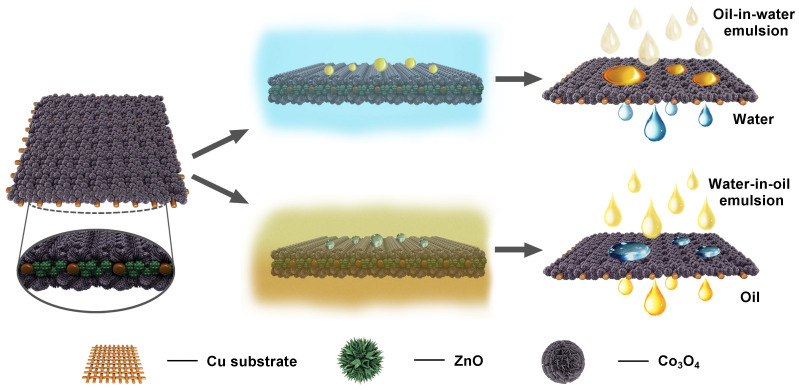
Schematic description. The structure of inorganic ZnO-Co_3_O_4_ overlapped membrane, switchable wettability when immersed in different media and the corresponding separation capacities of oil/water emulsions.
