# Supplementary material for: A Pure Inorganic ZnO-Co3O4 Overlapped Membrane for Efficient Oil/Water Emulsions Separation
Source: Sci Rep. 2015 Apr 22;5:9688. doi: 10.1038/srep09688 (PMC4405702; doi:10.1038/srep09688)
Supplement: Supplementary Information [file srep09688-s1.pdf]

Supplementary Information

A Pure Inorganic ZnO-Co_3O_4 Overlapped Membrane for Efficient Oil/Water Emulsions Separation

Na Liu [1†], Xin Lin[1†], Weifeng Zhang[1], Yingze Cao[1], Yuning Chen[1], Lin Feng[1]* & Yen Wei[1]

[1]Department of Chemistry, Tsinghua University, Beijing 100084, P. R. China. Correspondence and requests for materials should be addressed to L. F. (Email: fl@mail.tsinghua.edu.cn)

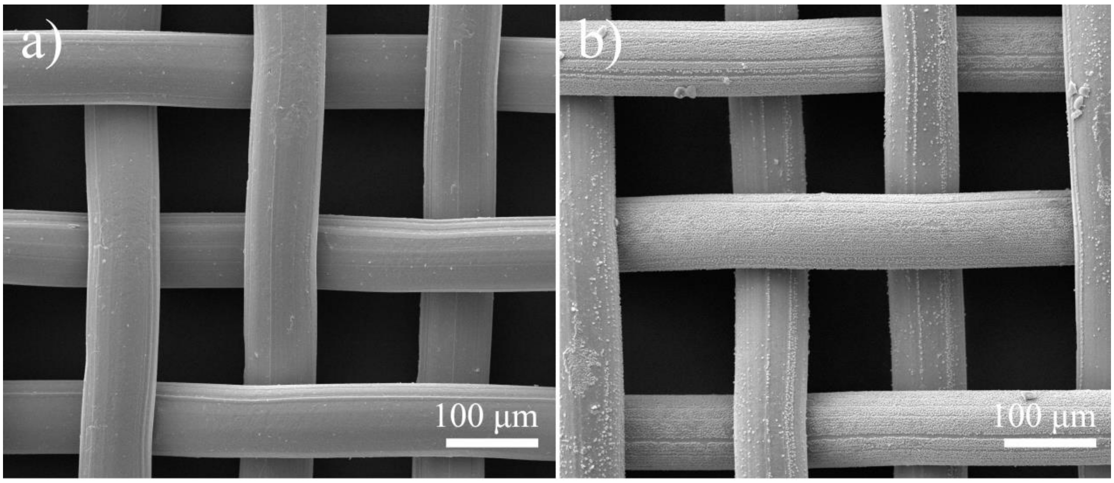

Supplementary Figure 1 | Morphology. (a, b) SEM images of Cu substrate before and after electro-deposition.

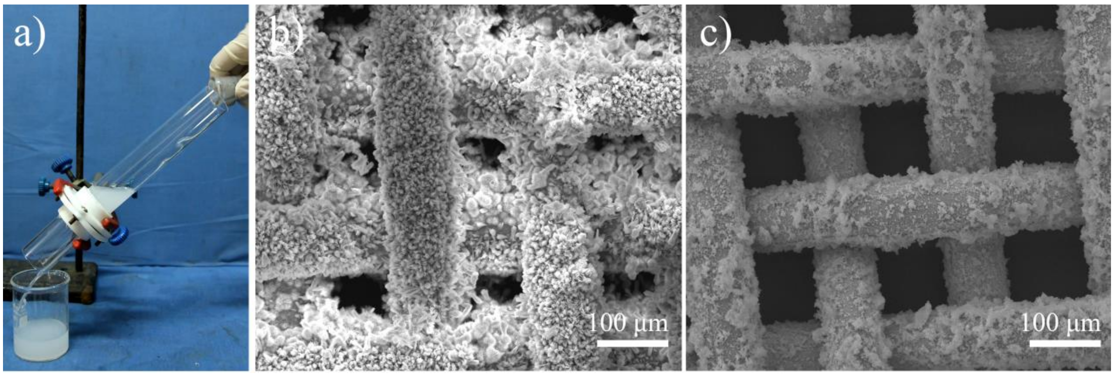

Supplementary Figure 2 | Separation test and morphologies of metal oxides. (a) Capacity test of the ZnO filled mesh for separating surfactant-free toluene-in-water emulsion. (b) SEM image of Co_3O_4 grown directly on copper mesh. (c) SEM image of TiO_2 grown directly on copper mesh.

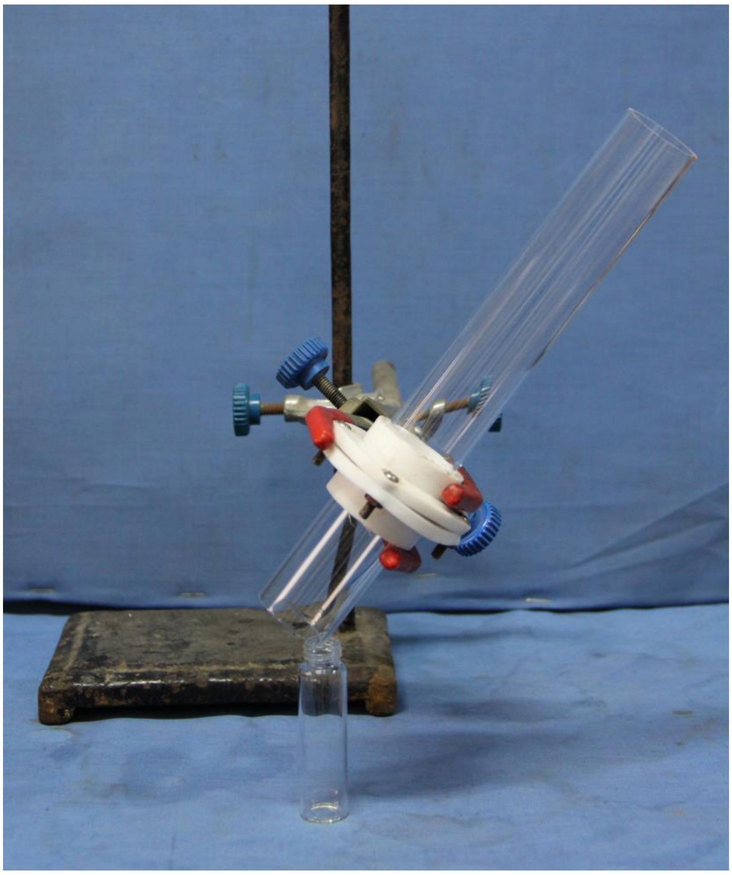

Supplementary Figure 3 | Separation device. The separation device is used in this work.

Emulsion Type	Surfactant-free Emulsions				Surfactant-stability Emulsions			
	Toluene/ Water	Octane/ Water	Gasoline/ Water	Diesel/ Water	Tween20/ Toluene/ Water	Tween20/ Octane/ Water	Tween20/ Gasoline/ Water	Tween20/ Diesel/ Water
Flux (L m^{-2} h^{-1})	232.9 ±64.1	197.7 ±43.0	129.9 ±15.1	235.1 ±49.8	66.9 ±2.4	88.6 ±0.4	43.5 ±4.4	24.6 ±4.2

Supplementary Table 1 | Flux comparison. The comparison of the fluxes between tween 20-free emulsions and tween 20-stabilized emulsions.

Pure Solvents	Water	Toluene	Octane	Gasoline	Diesel
Flux (L m^{-2} h^{-1})	~233.8	~718.6	~908.2	~826.4	~123.7

Supplementary Table 2 | Flux of pure solvents. The filterability of the membrane with pure water or pure oils.

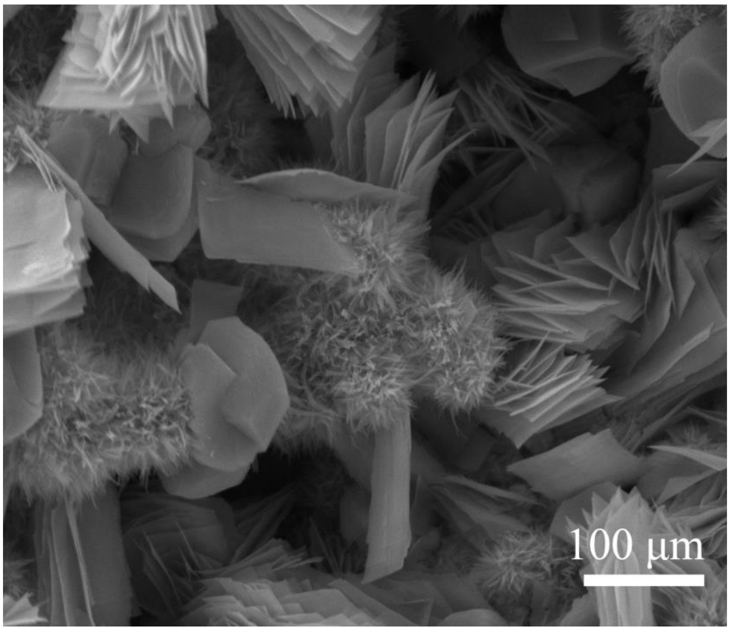

Supplementary Figure 4 | Reusability test. SEM image of the same membrane after fifty cycle filtrations.

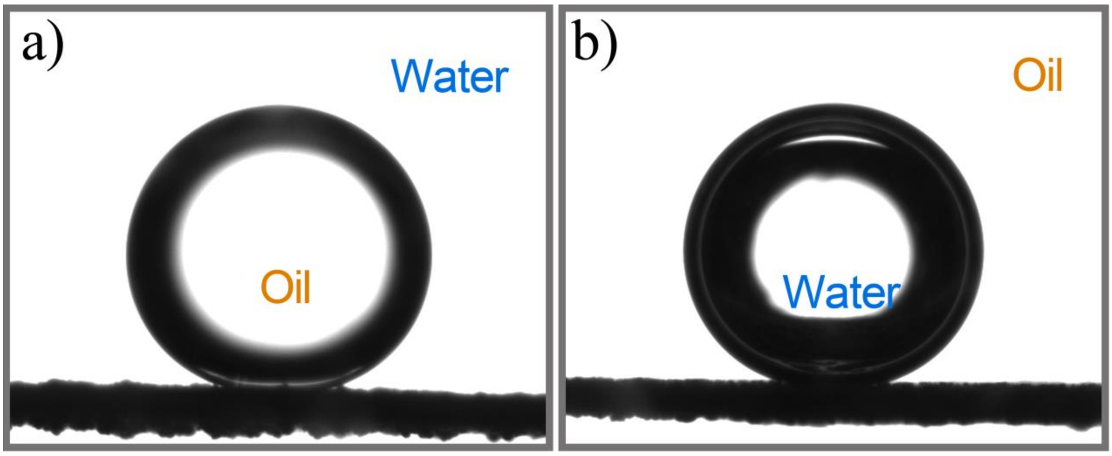

Supplementary Figure 5 | Thermal stability test. (a, b) Wetting behavior of the membrane toward 1, 2-dichloroethane in water and water in diesel after heated at 300 ^{o}C for 3h.
